# 

**Published:** 1912-10-19

**Authors:** 


### Answers to Correspondents.

"Norland Nurse."—As time is running short for becoming a member of an approved society it might be advisable for you to apply to the Prudential Society for Women (Holborn Bars), the National Amalgamated Approved Society (Euston Square), and the Hearts of Oak Society (Euston Road). The Nurses' Insurance Society regards hospital nurses only as eligible for membership.

### Buildings Contemplated and in Progress.

Airdrie.—The Town Council has ordered the preparation of a scheme for reconstructing the tuberculosis hospital.

Ballymena.—Application is being made to the Irish Local Government Board for sanction to a loan of £7,000 for a new infirmary.

Berkshire.—It is proposed to provide an isolation hospital for East Berks, and the county medical officer estimates the cost at £11,000.

Bishop Auckland.—Mr. R. B. Thompson is architect for additional blocks at Tindale Crescent and Helmington Row Hospitals. Tenders are being considered by the Joint Hospital Board.

Brighton.—The Corporation Committee has approved a scheme to purchase 1,072 acres of the Downs east of the borough boundary for £35,000 as a site for the convalescent home to be provided and endowed out of a gift of £40,000 to the town by Mr. John Howard.

Bromsgrove.—The Hospital Committee of the Rural District Council has decided to confer with Mr. A. Vernon Rowe regarding provision of a tuberculosis establishment.

Bury St. Edmunds.—A tender of £3,269 has been accepted for a new wing to the infirmary.

Chester.—It is proposed to erect a 150-bed sanatorium, to cost £25,000, to serve Cheshire county, the county boroughs of Chester, Birkenhead, Stockport, and Stoke-upon-Trent, also the borough of Wallasey.

Cornwall.—The Cornwall Sanitary Committee is submitting a sanatoria scheme to the Local Government Board for provisional approval.

Dalston.—£50,000 has been subscribed by German business men for improvements at the German Hospital, Dalston. Beds are to be increased in number from 125 to 150, two new theatres are to be added, and an out-patients' department will be instituted.

Devon.—The County Council is inquiring into the necessity for establishing hospitals for the following areas :—Holsworthy Urban and Holsworthy Rural Districts, Bideford Borough and Rural Districts, and Torrington Borough and Rural Districts.

Douglas.—The Isle of Man new hospital at Douglas, erected at a cost of £25,000 by the Trustees of the late H. B. Noble, Esq., has been formally opened. Mr. W. Henman was the architect.

Dumfries.—The nurses' home of the Dumfries and Galloway Royal Infirmary, erected at a cost of £2,500 has been formally opened. This is one item of the £12,000 reconstruction already referred to in our columns.

Dundee.—King's Cross Hospital is to be extended at a cost of more than £12,000; the works include a new pavilion.

Exeter.—Eleven tenders have been received for children's homes by the Guardians. The accepted one amounts to £6,359. Mr. R. M. Challice is architect to the Guardians.

Glamorgan.—The Pontypridd Guardians are considering a report on the combined scheme formulated by the various Boards of Guardians in Glamorganshire for establishing an institution for the care of the feeble-minded. The cost is estimated at £60,000.

Glenafton.—The Carrick District Committee has approved plans and estimate of £2,000 for hospital additions.

Hemel Hempstead.—Application for sanction to a loan of £8,000 has been made by the Joint Hospital Board for a new isolation hospital.

Hove.—At an estimated cost of £2,500 it is proposed to erect an additional ward block at the Borough Sanatorium.

Hull.—The Hull Sanitary Committee has ordered the preparation of plans for a permanent tuberculosis hospital, to cost not more than £15,000. Meanwhile temporary hospital and dispensary accommodation will be provided.—It is proposed to lay the foundation-stone of the new hospital for the Guardians very shortly; the buildings are estimated to cost close on £17,000.

Lancashire.—Mr. Charles H. Lancaster, F.S.I., is architect for the Alder Hey Hospital, which is being erected at a cost of about £100,000, exclusive of equipment.

### BUREAU OF INFORMATION.

### Rules for Correspondents.

1. Every letter, on whatever subject, must be accompanied by the coupon to be cut from the back cover (inside page) of The Hospital, current issue, and must contain the name and address of the correspondent with pseudonym for publication if desired.

2. Replies by post cannot be given, save under exceptional circumstances at the Editor's discretion.

3. Letters from Approved Homes in reply to special needs published in the Bureau should state terms and full particulars, and be sent prepaid under cover to the Editor of the Bureau with coupon and name enclosed for identification.

4. Proprietors of Homes which have not yet been entered on the List of Approved Homes, but have spare accommodation likely to suit special needs, are invited to write for an application form for registration. The fee for registration, which includes two announcements of the Home in the Bureau, is 5s.

5. Special needs of every description relating to those who are sick or in difficulties are made known in these columns free of charge.

6. All communications to be addressed to The Editor of The Hospital, 28 Southampton Street, Strand, London, W.C., and marked "Bureau of Information."

### Approved Homes.

Note.—*No Home is added to the List without direct medical and other testimony to its good management.*

**Brighton.**—114 Marine Parade, Kemp Town. Proprietress: Miss Janet Reilly. Medical, surgical, maternity, nerve, or permanent cases. Open situation near sea and downs. Wide and successful experience in nursing and in the general administration of a nursing home.

**Southampton.**—2 Rockstone Place. Principal: Sister Sarjantson. Medical and nerve cases. Dowsing Radiant Heat treatment. Electric and Swedish massage. Cottage by the sea as annexe.

### Training and Employment.

**Free Training for Male Masseur.**—The general rule about training is that if you do not pay in money you must pay in time. The National Hospital, Queen Square, Bloomsbury, receives men as probationers for two years' training, including massage, without premium; salary £10, £15. If you do not succeed in getting in there the best way to get trained would be to enlist as a soldier in the Royal Army Medical Corps. Candidates must be between the ages of eighteen and twenty-five, and be not less than 5 feet 3 inches in height. Apply at any military barracks for further particulars.—M. T. D.

**School for Domestics.**—We recommend your daughter to make application at any of the London hospitals for the post of wardmaid. You will find many such posts advertised in the nursing papers. We know of no other fully equipped School for Domestics among London institutions besides the one we have named.—T. C.

### Needs and Helpers.

**Work Cures.**—We fully concur with your opinion that a need exists for homes under trained nurses offering organised occupations to those mentally oppressed. The occupation might be purely manual, such as jam and confectionery making, gardening, or some of the many arts and crafts. Or it might be of a clerical character, such as typewriting or envelope directing. Needlework is not suitable except in special cases where great liking for it is shown, as it leaves too much opportunity for brooding. We have had a letter from one of our Approved Homes in which the experiment has been tried of allowing patients, if they desire it, to participate in the domestic work of the home. This is facilitated by having no ordinary domestics, and so organising the household that patients and nurses join in household duties. We are placing you in communication with various homes where an effort is being made to provide occupation for patients, and trust one of them may meet your needs.—Seeker (123a).

**Pensions for the Blind.**—We have ascertained that you could procure a pension within about a year's time of five shillings a month, to be raised later on to ten shillings a month, through the National Blind Relief Society. The address is the Rev. J. Pullein Thompson, The Vicarage, Tite Street, Chelsea, S.W. The condition, however, is that you get two new subscribers of one guinea each to nominate you. Do you think it would be possible for you to do this? We could perhaps help a little towards one subscription. Have you any friends in Maidstone who would help?—J. B.

### Nursing in the Empire and Abroad.

**An Opening at Suez.**—We understand that there is plenty of scope for a trained nurse working on her own account in Suez. When required they are at present sent for from Cairo, and as the train fare is £2 this extra expense is a great bar to getting British trained nurses. The usual fee is 10s. a day. Two resident doctors have expressed a wish for the presence of a trained nurse in the town, but there are several important points to be considered. Full training, with maternity certificate, would be indispensable. Next a knowledge of French is necessary, as a large number of French residents would be among the nurse's patients. Suez is not exactly an attractive place, living is expensive, and the most moderate hotel charges would be £2 a week. It would take a little time to form a connection, and the nurse who went out would need a little money in hand to start with. Later on, after getting a knowledge of the conditions prevailing, she could probably procure good rooms in the town at far less expense. In the opinion of a competent adviser, "the right kind of nurse would have much more work than she could undertake after a short time here. She would have to put work before everything else." In our judgment, two nurses, prepared to work together, well trained, not under thirty, with a facility in speaking French, and £50 at their disposal, would be more likely to succeed and be happy than one going out alone. We shall be pleased to put any nurses who satisfy the above requirements into communication with a correspondent on the spot, who will supply fuller information.

**Nurses for the War.**—There are absolutely no trained lay nurses whatever in Servia or Roumania, nor has there hitherto been the smallest demand for them. In Constantinople there are two trained nurses pretty constantly employed, and there is an English matron (Mrs. Fraser) at the British Seamen's Hospital, Constantinople. So far as nursing is concerned, the provision for the wounded is criticised in our Notes this week. But this does not prove that nurses would be permitted to go out, unless despatched through the regular channel of the Royal Red Cross.—Mercy.

**Copenhagen.**—Only nurses who hold a certificate from the Danish Red Cross Society are allowed to practice in Denmark. The regulation of the nursing profession in that country is very strict. There is no opening for foreigners, who would only be admitted to practice after undergoing the prescribed course of training in the country and passing the necessary examination. The Danish nurses are good linguists, and commonly speak English, French, or German.—Sister E. M.

### Letter-Box.

Mental Hospital.—If our correspondent will forward to us in confidence the name of the mental hospital referred to we will send down a representative and make an independent investigation.—C. N.

### Appointments.

Dr. Haddon, of Glasgow, has been appointed house surgeon at the Cameron Hospital, West Hartlepool.

Surgeon-General L. E. Anderson has been appointed Deputy Director of Medical Services, Irish Command.

Dr. H. E. Gamlen has been appointed *pro tem.* in charge of the *x*-ray apparatus at Hartlepools Hospital.

Mr. M. Rodger, M.D., has been appointed sixth assistant medical officer at Horton Mental Hospital in place of Mr. P. S. Vickerman, resigned.

The appointment of Dr. Cohen, resident medical officer at the Hull Workhouse, as senior tuberculosis medical officer to the Hull Corporation has been confirmed.

Mr. Archibald Donald, M.D., M.R.C.S., has been appointed to the Chair in Obstetrics and Gynæcology at Manchester, vacant through the death of Professor Sir William Sinclair.

Mr. Henry MacCormac, M.B.Edin., M.R.C.P., has been appointed to the newly created office of assistant physician to the department for diseases of the skin at the Middlesex Hospital.

Professor Lorrain Smith, M.A., M.D., having been appointed professor of pathology in the University of Edinburgh, has resigned the Chair of Pathology and Pathological Anatomy at Manchester.

The following appointments have been made at the Hampstead and North-West London Hospital: To be house physician, D. E. Fenwick, M.B., Ch.B.; and house surgeon, V. C. Vesselovsky, M.R.C.S., L.R.C.P.

The following appointments on the medical staff of St. Bartholomew's Hospital have been made: Mr. E. P. Cumberbatch, M.A., M.B., B.Ch., M.R.C.P., medical officer in charge of the electrical department; Mr. Hugh Walsham, M.D., medical officer in charge of the *x*-ray department; and Mr. R. C. Elmslie, F.R.C.S., surgeon in charge of the orthopædic department.

Mr. R. W. J. Pearson, fourth assistant medical officer at Claybury Mental Hospital, has resigned his position on appointment as second-class alienist in the service of the Egyptian Government. Mr. O. H. Bowen and Mr. P. S. B. Langton, the fifth and sixth medical officers, have been promoted to be fourth and fifth assistant medical officers, and Mr. E. G. T. Poynder has been appointed sixth assistant medical officer.

Sir Watson Cheyne, C.B., F.R.S., has been elected president of the Medical Society of London for the ensuing year.

### Gifts and Bequests.

Mr. Carnegie has laid the foundation-stone of a clinic for Dunfermline which is to cost £20,000.

The Treasurers of the Middlesex Hospital Cancer Charity have received another anonymous donation of £125.

The Chelsea Hospital for Women has received £52 10s. towards its Rebuilding Fund from Messrs. David Sassoon and Co.

The Committee for the removal of King's College Hospital to South London has received a cheque for 100 guineas from the Trustees of the late Mr. R. W. Edwards (fourth donation).

Mrs. Elizabeth Ann George, of Highgate, has bequeathed £500 each to the Royal Asylum for the Deaf and Dumb, London and Margate, St. Thomas's Hospital, the Hospital for Incurables at Streatham, the Great Northern Central Hospital, Holloway Road, N., and St. Leonard's Hospital, Sudbury.

The Secretary of the Coventry and Warwickshire Hospital has received an intimation that under the will of the late Miss Emma Maria Ward a legacy of £500 (less legacy duty) has been bequeathed to the institution. Under the will of Miss Ward's brother, who recently died, the hospital will also benefit by any surplus accruing after certain legacies have been paid.

Mr. Benjamin Grimes, of Leicester, has bequeathed a sum of £100 to Leicester Royal Infirmary. A cheque has been received for £119 6s., representing a year's contributions of the employees of Messrs. J. Gipson Clarke and Co. Grants of £20 12s. 4d. and £5 respectively have been made to the infirmary and Children's Hospital from the proceeds of the practice matches played by the Leicester Fosse Football Club.

Among recent contributions received for St. Bartholomew's Hospital towards the extinction of the debt of £57,000 are: Messrs. Thomas Wallis and Co., Ltd., £10 10s.; annual subscription and donations from Alderman and Sheriff C. A. Hanson, £251 17s. 7d.; Mr. Seth Taylor, £105; Mr. A. E. Baker, £100; Mr. Max Michaelis, £100; Mr. George MacAndrew, £50; Imperial Continental Gas Association, £50; Lord Trayner, £25; Mr. F. W. Hollams, £20; Anonymous, £20; and Mr. T. H. Mann, £20.

Mr. Charles Smith Fairbank, of Leamington, has bequeathed his residuary estate on trust for his wife for life, with remainder to his children or their issue in equal shares, and in the event of the failure of these trusts then £10,000 each to the General Hospital, Birmingham, and the Queen's Hospital, Birmingham; and the ultimate residue in equal shares to the Birmingham and Midland Free Hospital for Children, the Birmingham and Midland Hospital for Women, the Birmingham and Midland Eye Hospital, the Birmingham and Midland Ear and Throat Hospital, the Birmingham and Midland Homœopathic Hospital and Dispensary, the Royal Institute for Deaf and Dumb Children, the Royal Orthopædic and Spinal Hospital, the Birmingham Lying-in Charity, and the General Institution for the Blind.

Mr. Stuart de la Rue has forwarded £500 to the Royal Hospital for Diseases of the Chest, City Road, E.C., for the endowment of a cot.

Mr. Thomas Robert Johnston Logan, of Renfrewshire and Glasgow, ironmaster, has left £500 to the Victoria Infirmary, Glasgow, provided that the infirmary at the time of his decease is supported entirely by voluntary contributions, and is not aided by the State to any extent.

Mrs. Jane Clarke, of Howden, Yorks, has left £500 each to the Hull Royal Infirmary and the Leeds Royal Infirmary.

The Chelsea Hospital for Women has received for its Rebuilding Fund £52 10s. from the Prudential Assurance Company and £20 from the London County and Westminster Banking Company.

The Nelson Hospital and the North Wimbledon Cottage Hospital expect to receive considerably over £100 each from the proceeds of a cricket match played at the end of September between a Wimbledon Town team and a team containing many Surrey players. A bat inscribed with the names of the International teams playing in England this year was raffled, and itself realised more than £100. The winner then put it up for sale, and added some £20 to the proceeds of the raffle.

An anonymous lady has given, through the Matron, £120 for the purchase of *x*-ray apparatus at the Nelson Hospital, Wimbledon.

Surgeon-Major Bernard Kendall, retired, of Upper Norwood, a survivor of the Indian Mutiny, who died on September 3 last, aged eighty-one, left estate valued at £1,240 gross, with net personalty £1,126.

Surgeon-Major John Fitzgerald, of Co. Kerry, late of the Indian Medical Service, who died in June, aged seventy-five, left personal estate in the United Kingdom valued at £13,821, of which £7,291 is personal estate in England.

Sir Robert Mitton Hensley, of Putney, the oldest member and a former chairman of the Metropolitan Asylums Board, who died on August 5, aged seventy-two, left estate of the gross value of £18,048, of which the net personalty has been sworn at £15,408.

### Obituary.

Lieutenant-Colonel John William Jerome, R.A.M.C., retired, has died at South Norwood Hill. He was educated at King's College, and qualified M.R.C.S. in 1880. He was present at several actions during the South African War, in both the Cape and Orange River Colonies, and received the Queen's medal. Soon after becoming lieutenant-colonel in 1902 he retired.

Deputy Inspector-General George Kell, who has been on the Retired List since 1895, has died at Southsea. He entered the Navy in 1864, having qualified during the previous year. He saw service in many parts of the world, but he came particularly to the front in 1876, when he was on the staff of the Royal Naval Hospital at Haslar and received the thanks of the Admiralty for his treatment of the men who were injured through the explosion of a boiler on the battleship *Thunderer*. He received his medical education at Dublin.

Dr. George Garlick has died at Worthing as the result of an accident. He studied at University College, took his M.R.C.S. in 1874, and graduated M.D. at London University in 1876. His appointments included registrar at the Great Ormond Street Hospital for Children and clinical assistant at the Brompton. But children's diseases were his favourite study, and his various medical contributions include "Tubercular Peritonitis in Children," and "Ophthalmoscopic Observations in the Tubercular Meningitis of Children."

Dr. T. F. Gardner, of Bournemouth, has died very suddenly in the prime of life. His connection with Bournemouth dates back nearly thirty years, when he became resident medical officer of the Cottage Hospital there. This hospital eventually grew to the Royal Victoria Hospital, which is now amalgamated with the Royal Boscombe and West Hants Hospital—the process of combination being actively fostered by Dr. Gardner. Having settled in practice in Bournemouth he acted for many years as surgeon to the Royal Victoria Hospital, but later turned his attention more to the medical side of his profession and was appointed physician to the Royal Victoria and West Hants Hospital. Dr. Gardner held at one time the office of president of the Bournemouth Medical Society and of the Balneological and Clinatological Section of the Royal Society of Medicine. He studied at University College Hospital and qualified in 1883. He was a member of the Royal College of Physicians and M.D. of Durham University.

### THE BATH SPA EXPRESS.

A new service to Bath, specially designed for the comfort of visitors to that health resort, was inaugurated in October, when the first journey was made of the "Bath Spa Express." The express leaves Paddington every morning at eleven o'clock, accomplishing a non-stop journey to Bath in one hour and three-quarters. Seats may be reserved in advance, and a stewardess is carried to attend to the comfort of ladies and invalids travelling to Bath for the cure.

